# Glances at the Hospitals

**Published:** 1899-11-11

**Authors:** 


### GLANCES AT THE HOSPITALS.

### SUNDERLAND INFIRMARY.

This hospital has issued its one hundred and fifth annual report. Mr. Alderman Richardson is chairman of the General Committee, and Mr. Binns of the House Committee. The total number of patients treated during the year ended June 30th, 1899, was 7,754, being an increase of 521 on the previous year. Of the above number 2,647 were in-patients, and of this number 776 were children. Of the whole number of in-patients 1,519 were treated in the surgical wards, and 1,128 in the medical wards. The average daily number resident was 175, and the average cost per head was £3 2s. 4d. Or putting it another way the average stay in the hospital was 24 days, and the cost per bed occupied was £46 19s. We are glad to note that the bill for stimulants was not high. £48 for the whole year in a hospital containing an average of 175 patients must be looked upon as satisfactory, and it works out at an average of 4½d. per patient. The committee received during the year bequests and donations from various sources. For instance, £500 from Mr. H. T. Morton, £100 from Mr. A. Smith, £250 from Mr. Short, and £25 from Colonel Reed. The workmen's contributions were higher than in any previous year, and the total income reached £10,682. The nursing staff reaches a total of 47, being nearly one nurse to four patients; but 13 of the total were only probationers. It is evidence of the present popularity of the nursing profession that the Sunderland committee had 162 applications during the year from would-be probationers. Courses of instruction are given to the nurses. The silver medals were won by Nurses Wallis and Scott. The total number of operations performed during the year was 606, and chloroform was administered 594 times. The infirmary has a convalescent home situate at Hatherdene, near Harrogate.

### THE NORTH LONDON HOSPITAL FOR CONSUMPTION.

This hospital has issued, quite recently, its report for the year 1898. Among the patrons and vice-presidents are many names of those famous in all philanthropic movements—for instance, the Princess Christian, the Duchess of Grafton, the Duchess of Marlborough, the Duke of Norfolk, Earl Spencer, &c. Considering the great prevalence of consumption it is curious to reflect that there are so few special hospitals for it, more especially when it is known that so much depends on early treatment, and that patients have often to wait two months or more before they can hope for admission at some of our existing hospitals for chest diseases. This North London Hospital is situated near Hampstead Heath on very high ground, and is well removed from the germ-laden atmosphere of London. The hospital has done, and is doing, an immense amount of charitable work. It has treated 250,000 poor people from all parts of the kingdom, and last year it had 460 in-patients and 3,502 out-patients. There are 80 beds in the institution, but only 75 beds are open, as the resources of the hospital do not allow of the whole being used. The total expenditure is £7,000 per annum, and of this sum £5,000 has to be raised by donations and casual sources. We can readily believe the statement of the governors that help is urgently needed. Steps have been taken to carry out the open-air treatment of the disease, and one ward for men and another for women have been adapted for this purpose; but to carry it out as understood in the German consumption hospitals would, we should imagine, entail many alterations and the erection of properly constructed verandahs. A wing for 40 patients has been designed and will be carried out as soon as £5,000 have been received. One thousand pounds will endow a bed in perpetuity, £315 will confer the right to nomination to a bed during the life of the donor, and £52 10s. to nomination to a bed for one year. Game, fruit, flowers, and books are always acceptable. The office is at 41, Fitzroy Square, W., and all communications should be addressed to the secretary. Out-patients are also seen at 41, Fitzroy Square, on Mondays, Tuesdays, and Thusrsdays at 2 p.m.

